# The Structural Response of the Human Head to a Vertex Impact

**DOI:** 10.1007/s10439-023-03358-z

**Published:** 2023-09-21

**Authors:** Darcy W. Thompson-Bagshaw, Ryan D. Quarrington, Andrew M. Dwyer, Nigel R. Jones, Claire F. Jones

**Affiliations:** 1 School of Electrical & Mechanical Engineering, The University of Adelaide, Adelaide, SA 5005 Australia; 2 Clinical and Research Imaging Centre, South Australian Health and Medical Research Institute, Adelaide, SA 5000 Australia; 3 Centre for Orthopaedic & Trauma Research, Faculty of Health and Medical Sciences, The University of Adelaide, Adelaide, SA 5005 Australia; 4 Department of Orthopaedics & Trauma, Royal Adelaide Hospital, Adelaide, SA 5000 Australia; 5 Adelaide Medical School, The University of Adelaide, Adelaide, SA 5000 Australia

**Keywords:** Head-first impact, Stiffness, Biomechanics, Compression, Skull fracture, Cephalus

## Abstract

In experimental models of cervical spine trauma caused by near-vertex head-first impact, a surrogate headform may be substituted for the cadaveric head. To inform headform design and to verify that such substitution is valid, the force-deformation response of the human head with boundary conditions relevant to cervical spine head-first impact models is required. There are currently no biomechanics data that characterize the force-deformation response of the isolated head supported at the occiput and compressed at the vertex by a flat impactor. The effect of impact velocity (1, 2 or 3 m/s) on the response of human heads (*N* = 22) subjected to vertex impacts, while supported by a rigid occipital mount, was investigated. 1 and 2 m/s impacts elicited force-deformation responses with two linear regions, while 3 m/s impacts resulted in a single linear region and skull base ring fractures. Peak force and stiffness increased from 1 to 2 and 3 m/s. Deformation at peak force and absorbed energy increased from 1 to 2 m/s, but decreased from 2 to 3 m/s. The data reported herein enhances the limited knowledge on the human head’s response to a vertex impact, which may allow for validation of surrogate head models in this loading scenario.

**Supplementary Information:**

The online version contains supplementary material available at 10.1007/s10439-023-03358-z.

## Introduction

High-energy near-vertex head-first impacts, such as those that occur during automotive and sports accidents, can cause devastating cervical spinal injuries that are often associated with tetraplegia [22, 26]. Some experimental studies have investigated the mechanisms of cervical trauma resulting from near-vertex head-first impacts using cadaveric head-neck specimens [17, 19], while others have substituted the cadaveric head with a headform [11, 16, 23, 24]. Surrogate headforms (e.g., Hybrid-III 50th percentile male) allow researchers to isolate the response of the cadaveric neck, and to apply boundary conditions that are challenging to achieve with a cadaveric head-neck specimen. To ensure that such headform-neck systems maintain biofidelity throughout the multiphase loading that occurs during head-first vertex impacts [18], the impact response of the isolated surrogate head must mimic that of cadaveric heads. However, no previous studies have characterized the mechanical response of isolated heads with neck-end boundary and loading conditions that appropriately replicate that experienced by the head during a near-vertex head-first impact.

**Fig. 1:**
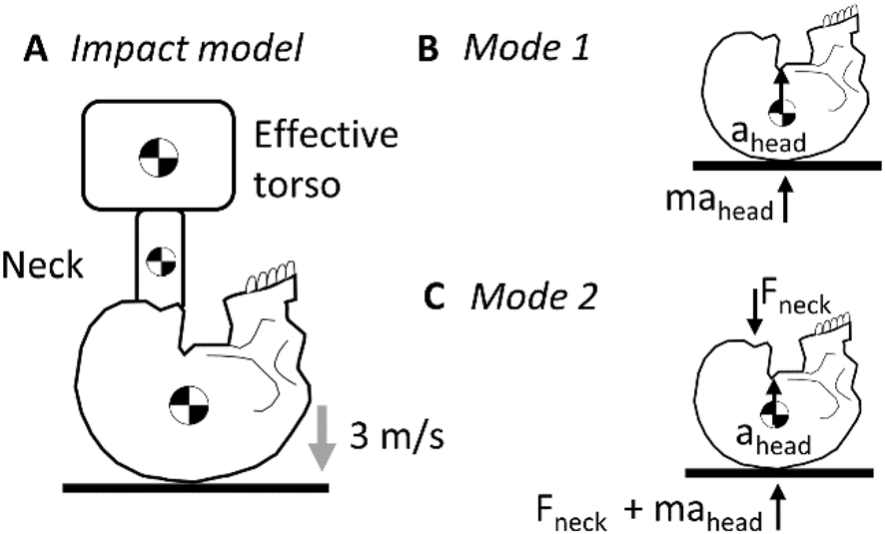
**A** Cervical trauma resulting from dynamic axial compression has previously been investigated using a head-first impact model, with a 3 m/s impact velocity and 16 kg effective torso mass, representing the upper torso of a 50th percentile male [17]. During the impact event, two modes of head and neck loading are observed. In the first mode **B**, the head is subjected to its own inertial force, given by the product of its mass (*m*_head_) and acceleration (*a*_head_). In the second mode **C**, the head is subjected to its own inertial force plus the reaction force from the neck (*F*_neck_) [15]

Clinically relevant cervical spine trauma has been reliably produced in ex vivo head-first impact models with a 16 kg upper torso mass (50th percentile male) and an impact velocity of ~ 3 m/s (Fig. 1A) [17]. These models have demonstrated that the head and neck loading response to near-vertex head-first impacts is initially decoupled. Upon impact, the head resists its own inertia during contact with the external rigid surface (mode 1; Fig. 1B). The following torso then compresses the entire head-neck system against the external surface (mode 2; Fig. 1C), during which cervical spine injuries and skull base fractures can occur [15]. In such cadaveric head-neck models, the temporal kinetic response of the neck, and the incidence of cervical spine injuries, are sensitive to the system compliance at the head end (i.e. padded versus rigid impact surface) [19]. When a surrogate headform was substituted for the cadaver head in similar models, the temporal kinetic response of the headform-neck did not consistently mimic the response of cadaveric head-necks [16, 23], potentially due to non-physiological compliance of the headform. To achieve a biofidelic neck loading response in near-vertex head-first impact models that exclude the cadaver head, the compliance of the surrogate headform should mimic that of the average human head.

To ensure biofidelity, the force-deformation response of surrogate headforms should lie within the cadaveric response corridor (mean ± standard deviation) for equivalent loading conditions [21]. It is important that surrogate headforms are validated for specific loading scenarios, as the human head’s force-deformation response is dependent on the input energy (velocity and mass) [13, 27, 28], anatomical region of contact [13, 27, 28] and impactor surface (flat versus curved, large versus focal) [25, 27, 30]. Considering a near-vertex head-first impact, the isolated response of the head may differ between mode 1 (vertex compression due to head mass and mode 2 (vertex and occiput compression due to torso mass) due to the change in regions of loading.

Two studies have characterized the force-deformation response of the head to vertex impacts. Loyd et al. [13] performed freefall drop tests of cadaveric heads onto the vertex, replicating mode 1. Yoganandan et al. [28] applied axial compression loading (7–8 m/s) to the vertex of isolated head, via a hemispherical anvil, while supporting the entire skull base (occiput, temporal and maxilla). As the loading and boundary conditions of the latter study are not equivalent to those typically implemented for near-vertex head-first impact neck trauma models (~ 3 m/s impact velocity, with a large, flat impactor), those data may not accurately represent the force-deformation response of the isolated head in mode 2.

The aim of the current study was to determine the force-deformation response corridors of the head, while supported at the occiput, and compressed by a flat impactor at the vertex, at 1, 2 and 3 m/s, and to assess the effect of impact velocity on the peak force, peak deformation, stiffness, and absorbed energy.

## Materials and Methods

Institutional Human Research Ethics Committee approval was granted for this study (Reference No. H-2018-261).

### Specimen Preparation

Twenty-two fresh-frozen cadaveric heads (76 ± 14 years, 13 male) were used for the study following review of pre-test high resolution computed tomography (CT) scans (Biograph64, Siemens, Munich, Germany; 120 kV, 179 mAs, 512 x 512 resolution, 0.6 mm slice thickness; reconstructed with voxel sizes of 0.488 × 0.488 × 0.3 mm). Specimens were screened for existing head trauma and disease by a radiologist (AMD) or neurosurgeon (NRJ) and excluded if cranial vault fractures or other abnormalities were identified. Two specimens with existing minor facial trauma (nondisplaced, < 8 mm in length; ID 4 and 6) were included in the study.

Anthropometric measurements were obtained from the pre-test CT scans using image segmentation software (MIMICS v24, Materialise NV, Leuven, Belgium). The CT scans were density-calibrated using an electron density calibration phantom (Model 062, Computerized Imaging Reference System, Virginia, USA). The following measurements were obtained from CT images: bone mineral density (BMD) and skull thickness at the vertex (apex of the sagittal suture), and the cranial height (basion to bregma), cranial length (glabella to the most posterior point) and cranial width (porion to porion).

Cephalic-cervical spine specimens were stored at − 20 °C prior to use, and were thawed at 4 °C for 36 h prior to preparation, ensuring all tissues were completed thawed. Tests were performed at room temperature (21 ± 1 °C). Specimen hydration was maintained throughout storage, preparation, and testing by wrapping in phosphate buffered saline (PBS) soaked gauze, and spraying with PBS. Specimens were prepared by disarticulating the neck and spinal cord at the occipital condyles, leaving the intracranial contents intact, and removing the mandible and associated soft tissue. The bony surface of the occipital condyles, clivus and foramen magnum were cleared of soft tissue. The isolated head mass (without mandible) was measured (0.01 kg resolution; UM-501, Tanita, Perth, Australia).

**Fig. 2:**
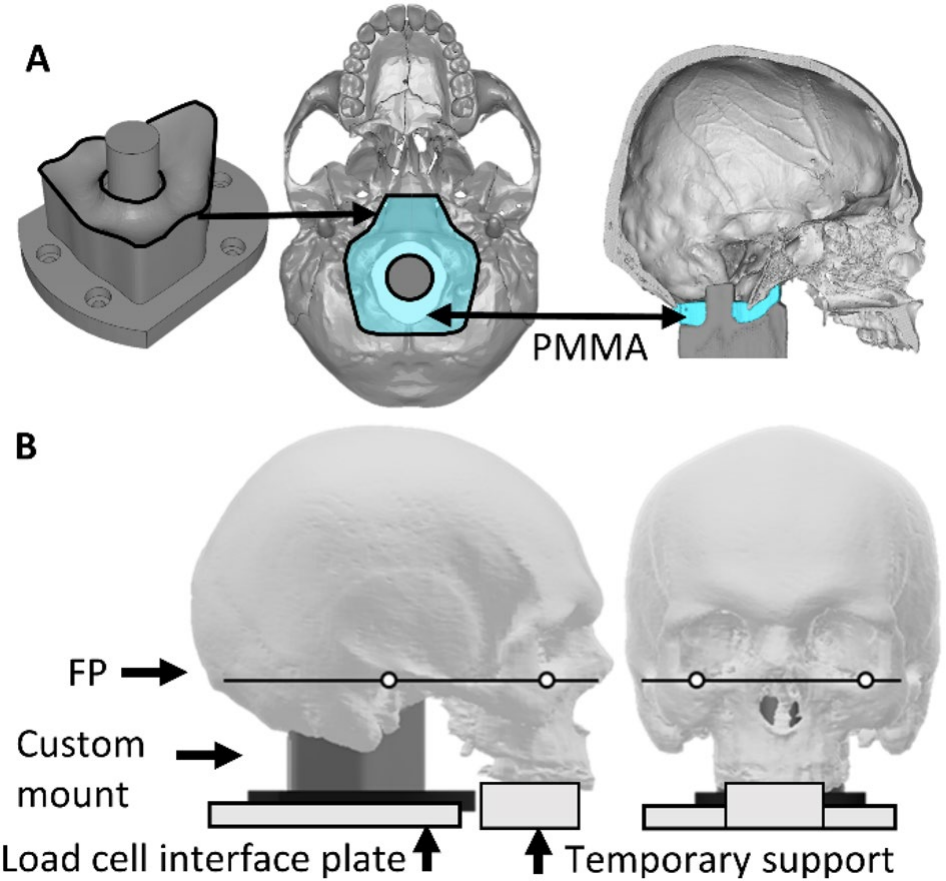
**A** A custom 3D-printed mount was designed to support the specimens at the occipital condyles, clivus and the perimeter of the foramen magnum, as well as provide a means to attach the specimen to a load cell. The mount interface geometry was derived from 3D models of the skull base. A layer of polymethylmethacrylate (PMMA; blue region) was applied to the mount surface prior to positioning the cadaveric heads on the mount. **B** Bilateral Frankfort plane (FP) landmarks (porion and orbitale) [9] were aligned with a horizontal laser and the head was temporarily supported until the PMMA cured

Specimens were rigidly supported at the skull base by a custom, 3D-printed, support mount (Fig. 2A). The mount geometry was designed to approximate the surface of the occipital condyles, clivus and the foramen magnum perimeter, which was determined from 3D models of the skull derived from CT images. The mounts were 3D-printed (Zortrax, Olsztyn, Poland; 100% infill) in polyethylene terephthalate glycol filament (PETG); their stiffness (39,304 ± 8382 N/mm) was evaluated in additional testing (as detailed in Supplementary Material) and deemed to be sufficiently larger than the stiffness of the head. A layer of polymethylmethacrylate (PMMA; Kulzer, Hanau, Germany) was applied to the bony surfaces that would be in contact with the mount to provide congruency between the bony surface and generalized mount surface, and to secure the specimen to the mount with a consistent orientation. The head was pressed onto the mount, then the Frankfort plane landmarks [9] (augmented with markers) were aligned with a horizontal laser level (TCL-1XR, General Titanium Series, Geelong, Australia), in the sagittal and coronal planes. The head was temporarily supported until the PMMA cured (Fig. 2B).

### Mechanical Loading

**Fig. 3:**
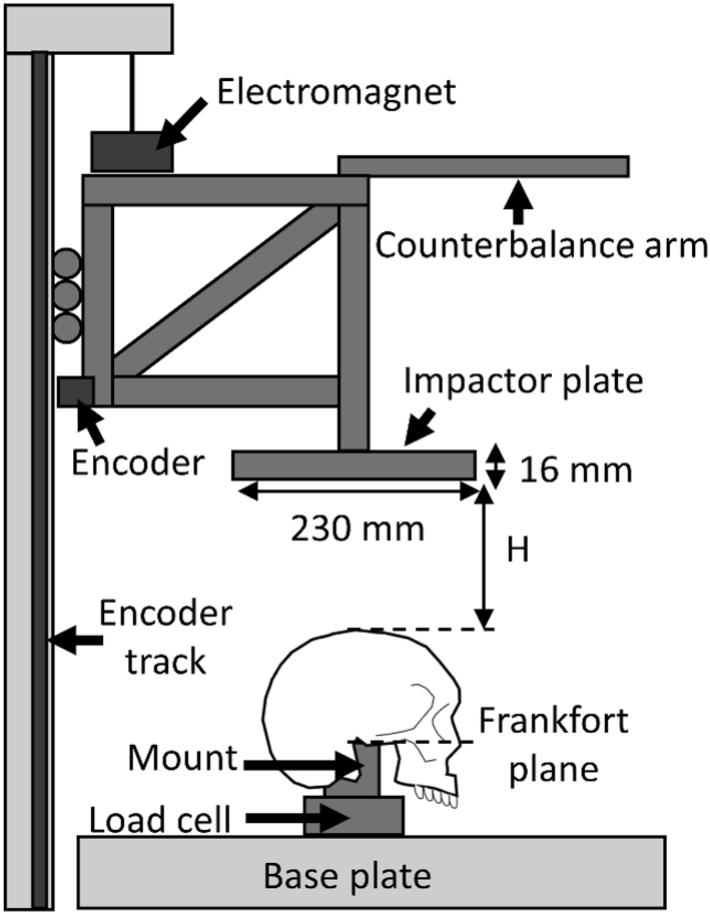
The specimen-mount assembly was fixed to a load cell at the base of a custom drop tower. The magnetic encoder, fixed to the carriage, measured displacement along the track. The combined mass of the carriage and its attachments (impactor plate and counterbalance arm) was 16 kg, to represent the upper torso of the 50th percentile male. The weighted carriage was suspended by the electromagnet at a predetermined height (*H*), relative to the vertex, to achieve the intended impact velocity (1, 2 or 3 m/s)

The specimen-mount assembly was fixed to a six-axis load cell (K6D110 ± 20 kN, ME, Hennigsdorf, Germany) at the base of a custom drop tower (Fig. 3). A 16 kg carriage (50th percentile male upper torso mass [17]), with a flat aluminum impactor plate was fixed to a vertical linear rail. The carriage was released by an electromagnet from predetermined heights (relative to the vertex of the specimen) to impact each specimen once, at 1, 2 or 3 m/s. The carriage was free to rebound, and its motion continued until it came to rest on the specimen.

### Instrumentation and Data Collection

Carriage position was measured using the incremental displacement output of a linear magnetic encoder (±10 µm resolution; LM15, Rotary and Linear Motion Sensors, Komenda, Slovenia). Loads and carriage position data were collected at 50 kHz using a data acquisition system (PXIe-1073, BNC-2120, and PXIe-4331, National Instruments, USA) and custom LabVIEW code (2019, National Instruments, Texas, USA). Two high speed cameras (i-Speed TR, Olympus Corporation, Tokyo, Japan) recorded frontal and right lateral views of the impact at 5 kHz. The data acquisition system sent an external trigger voltage to the cameras and electromagnet, which released the carriage, to synchronize data capture.

### Data Processing

**Fig. 4:**
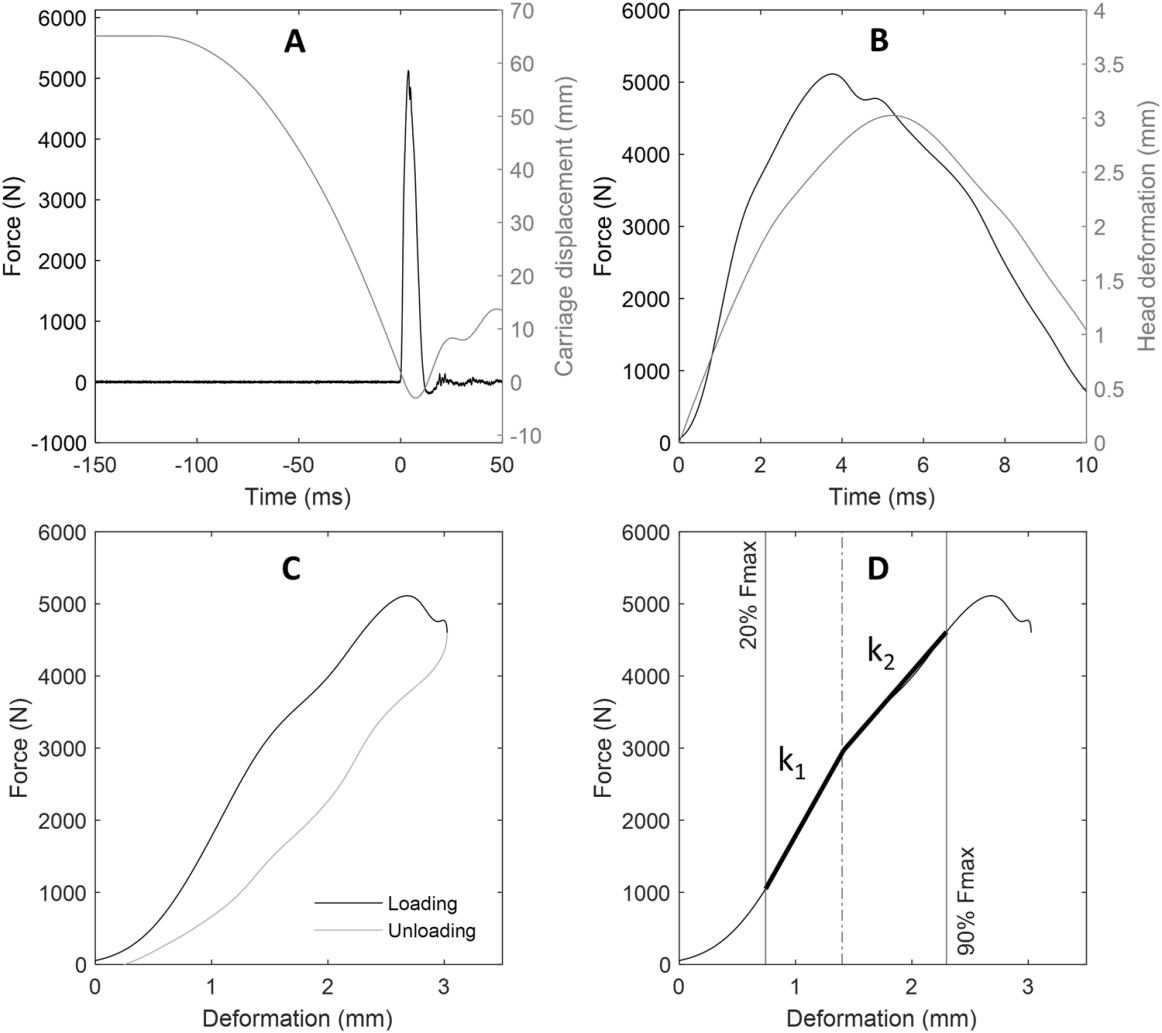
Exemplar force-time, deformation-time and the resultant force-deformation response for a 1 m/s impact (ID: 4). **A** Force-time (black) and carriage displacement-time (gray) from the point of carriage release until just after impact. **B** Extracted force-time (black) and deformation-time (gray) from the onset of contact (50 N increase) until the carriage returned to the same position. **C** The loading (black) and unloading (gray dashed) force-deformation response. **D** Bilinear approximation of a bilinear force-deformation response between 20 and 90% of the maximum force. The effective stiffness was taken as the average of the two stiffness values (*k*_1_ and *k*_2_)

Data were processed using custom MATLAB code (R2020a, Mathworks, Massachusetts, USA). A second-order, two-way, low-pass Butterworth filter, with a 4 kHz cut off frequency was applied to the data. Force-time and displacement-time data (Fig. 4A) were extracted from the onset of contact (50 N increase in force) to peak deformation or failure (whichever occurred first). Fracture was identified by a sudden reduction in force and was verified against the time-synchronised high-speed video images. Head deformation was evaluated as the displacement of the carriage during the defined loading region (Fig. 4B). The resulting force-deformation responses up to peak deformation/failure (Fig. 4C, D) were used for further analysis, and data beyond the loading region were not further processed. Absorbed energy was defined as the area under the force-deformation response. Stiffness was defined as the slope of the force-deformation response; however, separate methods were employed for linear and bilinear responses. For specimens that demonstrated a linear force-deformation response, stiffness was calculated as the slope of a linear regression fit between 20 and 90% of the peak force. For specimens that demonstrated a bilinear force-deformation response (Fig. 4D), a bilinear approximation was evaluated between 20 and 90% peak force using the MATLAB tool, slmengine [4]. To facilitate statistical comparisons, an effective stiffness was evaluated as the mean of the two slopes, *k*_1_ and *k*_2_, such that each region contributed equally to the mean regardless of their relative contribution to total deformation. The regional stiffness’ (*k*1, *k*2) are reported (Supplementary Table S1).

Force-deformation response corridors were generated for each impact velocity using the box method [12]. Firstly, the characteristic average force-deformation response was generated. Then, the standard deviation for force (± vertical variation) and deformation (± horizontal variation) at each point along the characteristic average response was calculated; these defined four variation points associated with each point along the characteristic average. The response corridor was evaluated as the region bounded by the extreme points along the characteristic average.

### Post-test Specimen Evaluation

After the test, a region of scalp anterior to the impact site (at the bregma) was resected and scalp thickness was measured using Vernier callipers (0.01 mm resolution; Absolute, Mitutoyo, USA). The skull was visually inspected for fractures at the impact site and around the mount. Post-test CT scans (as above) were obtained to assess the specimen and mount condition. For specimens with a pre-test facial fracture (IDs 4 and 6), comparison of pre- and post-test scans confirmed there was no detectable propagation of the pre-existing fractures. No external or internal damage was observed on or inside the mounts.

### Statistics

Four generalized linear models (GLM) were developed using SPSS (v27, IBM, Illinois, USA) to assess the effect of impact velocity on peak force, peak deformation, stiffness, and absorbed energy. Each model was adjusted for factors that may have influenced the loading response of the tissue, including: sex, age, head mass, scalp thickness, vertex bone thickness and BMD, and the cranial height, length, and width. The effect of each adjustment factor, when in the presence of impact velocity, was individually assessed against each outcome measure. Adjustment factors that demonstrated some statistical association (*p* < 0.2) [10] were included in the initial multivariable GLM, which was then iteratively refined using a backwards step-wise approach until only significant factors remained (*α* = 0.05). Post hoc analyses with Bonferroni corrections were conducted to assess significant differences between impact velocities. The descriptive statistics are presented in text (mean ± standard deviation), and GLM outcomes are presented in the Supplementary Material (Supplementary Tables S2, S3).

## Results

**Fig. 5:**
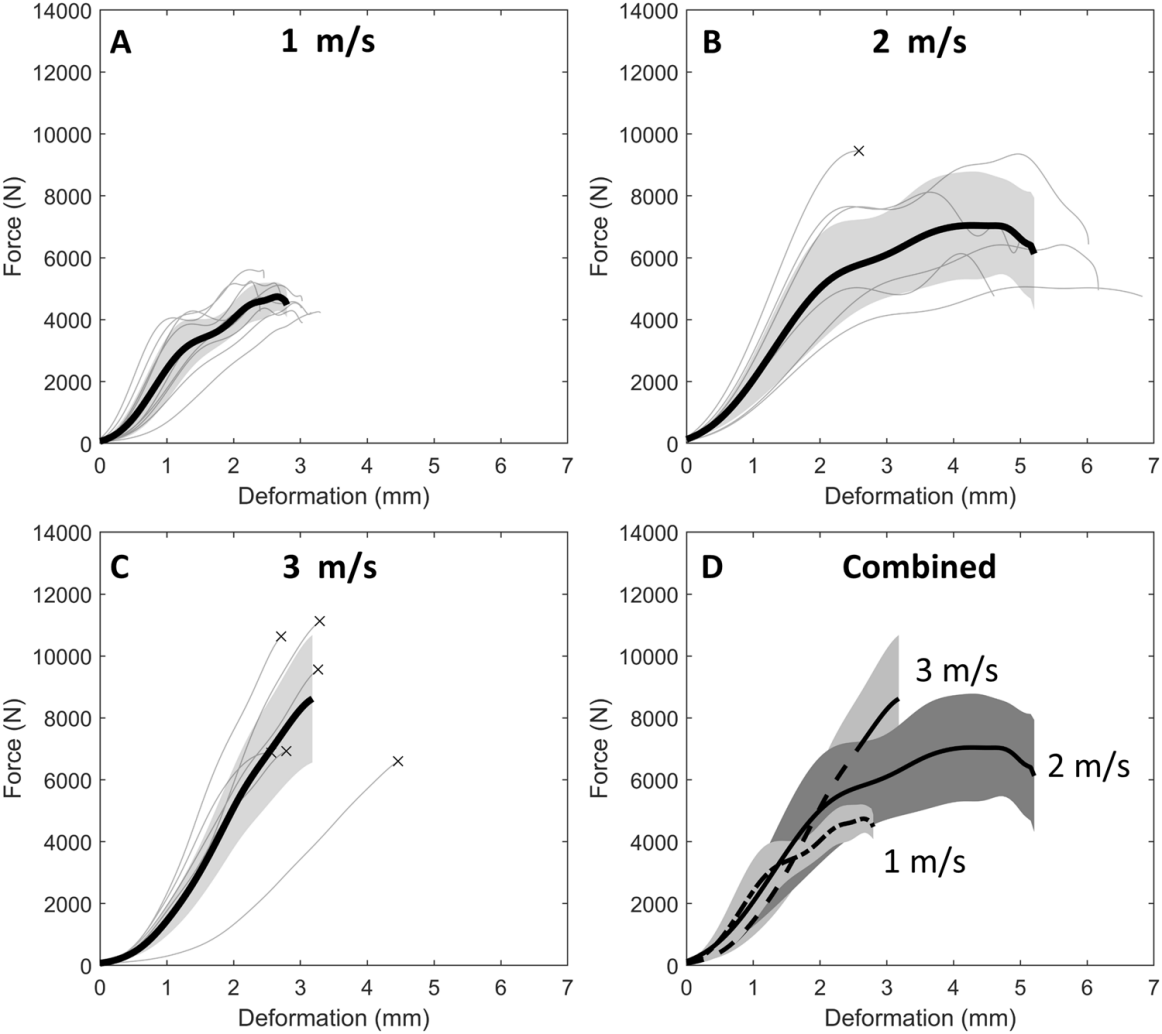
**A**–**C** Specimen force-deformation responses for 1, 2 and 3 m/s impact velocity. **D** Overlaid characteristic average force-deformation curves for each impact velocity. The shaded regions indicate ± 1 standard deviation. ^x^ Specimen fractured due to impact

The force-deformation relationships across all impact velocities exhibited a non-linear response that consisted of an initial toe-region, then either a bilinear or linear response (Fig. 5A–C). A bilinear response was observed for all specimens that did not fracture, whereas a linear response was observed for all specimens that fractured. All fractures occurred in the posterior fossa of the skull base, outside of the area in which the PETG/PMMA mount contacted the bone (Supplementary Table S4; Figure S3). The characteristic average response corridors for all impact velocities overlapped during the toe-region and first linear region but diverged during the second linear region for the 1 and 2 m/s groups (Fig. 5D).

**Fig. 6:**
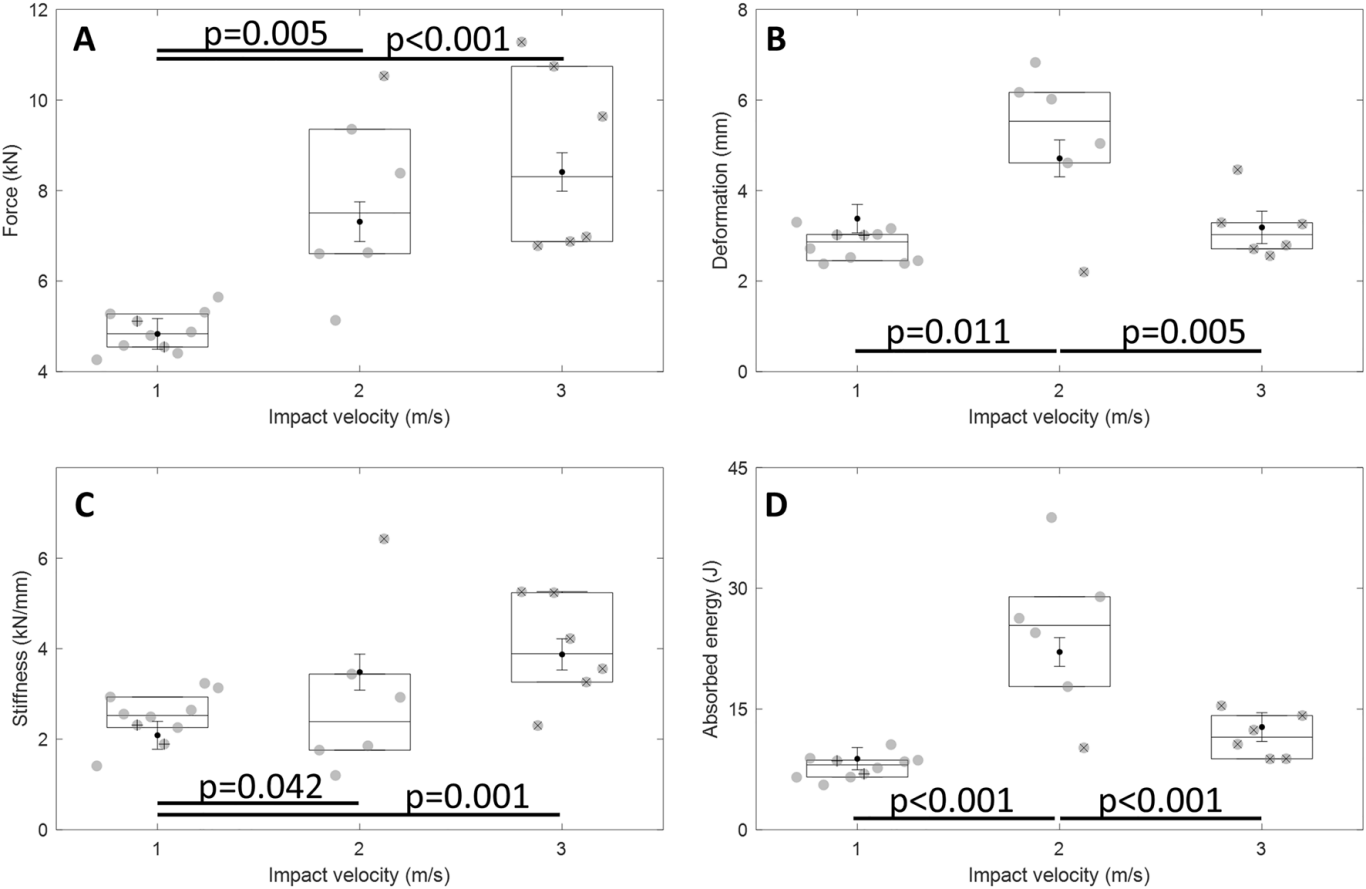
Box plots (mean, interquartile range) for **A** peak force, **B** peak deformation, **C** stiffness and **D** absorbed energy, for each impact velocity. Grey circles indicate measured data points. Black circles with error bars indicate estimated marginal means and standard errors from the final generalized linear models. Significant differences indicated between impact velocities for each outcome measure correspond to Bonferroni-adjusted post hoc comparisons. ^x ^Specimen fractured due to impact. ^+^Specimens with an existing fracture (1 m/s: ID 4 and 6)

The heads withstood peak forces ranging between 4261 and 9354 N, predominantly without fracturing at 1 and 2 m/s, but consistently fractured at 3 m/s at forces ranging between 6781 and 11281 N (Table 1). The effect of impact velocity on peak force was dependent on cranial height (*p* = 0.03). Peak force increased from 1 to 2 and 3 m/s, but there was no difference detected between 2 and 3 m/s (Fig. 6A). Head deformation was influenced by impact velocity (*p* = 0.003) when adjusting for BMD (*p* = 0.011), increasing from 1 to 2 m/s, but decreasing from 2 to 3 m/s (Fig. 6B). The relationship between stiffness and impact velocity was dependent on cranial height (*p* < 0.001) and BMD (p < 0.001). The heads exhibited a stiffer response at 2 and 3 m/s, compared to 1 m/s, but there was no difference detected between 2 and 3 m/s (Fig. 6C). Absorbed energy was influenced by impact velocity (*p* < 0.001). The heads absorbed more energy from 1 to 2 m/s, but from 2 to 3 m/s they absorbed less energy (Fig. 6D).

**Table 1:** Specimen demographics, anthropometric measurements, and the four outcomes—peak force, peak deformation (*D*), stiffness and absorbed energy

ID	Velocity (m/s)	Sex	Age	Head mass (kg)	BMD (kg/m^3^)	Head dimensions (mm)					Peak force (N)	Peak D (mm)	Stiffness (N/mm)	Energy (J)
						ST	BT	Width	Length	Height				
1	1.07	M	81	2.9	1554	6.4	6.1	118.7	186.7	145.5	4261	3.30	1410	6.52
2	1.09	F	105	2.3	1628	1.9	5.8	104.6	188.9	139.2	5274	2.72	2933	8.88
3	1.07	F	83	2.9	1787	2.8	6.4	119.3	198.2	135.2	4577	2.38	2555	5.57
4^a^	1.07	M	73	3.9	1736	7.7	6.1	128.9	207.3	147.8	5112	3.02	2312	8.56
5	1.08	F	55	2.7	1687	3.8	6.2	104.9	177.0	143.8	4798	2.52	2493	6.54
6^a^	1.07	M	65	4.0	1665	7.0	6.7	125.0	209.6	154.1	4543	3.01	1892	6.94
7	1.07	F	55	2.9	1785	5.5	5.7	111.5	195.3	139.7	4405	3.03	2257	7.68
8	1.07	M	64	3.2	1625	2.7	4.2	114.1	215.9	148.3	4875	3.16	2644	10.58
9	1.07	M	95	3.4	1800	1.1	5.7	99.7	179.8	131.7	5310	2.39	3235	8.46
10	1.07	M	67	2.6	1758	1.9	6.1	112.8	182.7	140.3	5645	2.45	3134	8.64
Mean	1.07	–	74	3.1	1703	4.1	5.9	114.0	194.1	142.6	4881	2.80	2487	7.84
SD	0.01	–	17	0.6	87	2.4	0.7	9.3	13.4	6.7	447	0.35	558	1.47
11	2.06	M	63	3.4	1591	4.2	5.1	128.2	190.2	154.1	6604	6.17	1758	22.35
12	2.06	F	90	3.0	1482	5.2	6.6	97.6	187.3	135.2	5132	6.83	1200	24.49
13	2.06	F	83	3.3	1547	2.3	7.0	108.4	196.5	149.5	9354	6.02	3438	34.17
14	2.01	F	83	2.4	1481	3.3	8.3	111.0	179.3	140.9	6628	4.61	1853	17.79
15	2.03	M	74	3.3	1686	5.9	7.8	106.7	196.4	152.9	10532^b^	2.20	6427	10.18
16	2.01	M	59	3.1	1610	2.2	6.8	107.7	195.3	150.6	8383	5.04	2925	23.58
Mean	2.04	–	75	3.1	1566	3.8	6.9	109.9	190.8	147.2	7772	5.15	2934	22.09
SD	0.02	–	12	0.4	79	1.8	1.1	10.1	6.8	7.5	2007	1.65	1898	7.39
17	3.10	M	81	3.1	1606	4.4	4.7	126.8	188.4	152.3	11281^b^	3.46	4657	17.31
18	2.95	F	91	2.7	1626	2.9	7.1	115.6	177.6	139.2	6781^b^	4.73	2123	12.43
19	3.08	M	69	3.3	1715	3.5	7.2	120.6	191.9	150.6	10744^b^	2.82	5010	13.56
20	3.09	M	71	3.1	1629	1.9	6.0	122.1	186.7	148.9	6873^b^	2.50	4106	8.40
21	3.09	M	99	2.9	1614	2.9	5.4	127.0	189.6	140.3	6978^b^	2.90	3185	9.59
22	3.06	F	66	2.4	1552	2.5	6.8	112.3	173.5	143.8	9637^b^	3.38	3468	15.30
Mean	3.06	–	80	2.9	1624	3.0	6.2	120.7	184.6	145.9	8716	3.30	3758	12.77
SD	0.05	–	13	0.3	53	0.9	1.0	5.9	7.3	5.5	2083	0.79	1057	3.38

*BMD* bone mineral density, *ST* scalp thickness, *BT* bone thickness, *SD* standard deviation

^a^Specimens with pre-existing facial fractures

^b^Fracture force

## Discussion

Surrogate headforms have been incorporated into ex vivo models of near-vertex head-first impact in an attempt to isolate the mechanical response of the cadaveric cervical spine, as they may reduce some of the variability associated with impacting cadaveric heads. The kinetic response of the cadaveric neck is sensitive to head-end compliance [19], so it is important to validate surrogate headforms’ response with cadaveric data in equivalent loading scenarios. In the current study, cadaveric heads were subjected to vertex impacts from a 16 kg carriage (simulated 50th percentile upper torso mass) while constrained by a custom rigid occipital support. Impact velocity significantly affected the loading response of the head, and influenced the production of skull base ring fractures. These results may aid validation of surrogate headforms for applications in near-vertex head-first impact models, which may be used to further improve the understanding of cervical spine trauma due to a head-first impact.

**Fig. 7:**
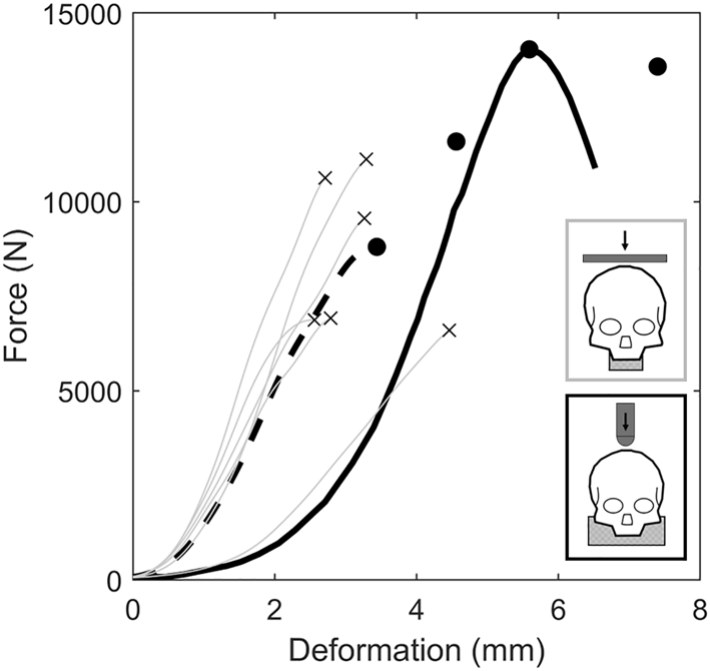
The force-deformation response of the human head when subjected to destructive vertex impacts, while supported at the occiput. The 3 m/s specimen responses (grey), with corresponding point of fracture (times), and the characteristic average from the present study (dashed black line). An exemplar response (black line; ID 7; extracted using GRABIT [6]) and the points of failure (black circle; IDs: 7, 9, 11, 12) from Yoganandan et al. [28] (7–8 m/s)

For 3 m/s impacts, the loading response and fractures produced differed from the only previous study to dynamically compresses the head at the vertex and occiput, simultaneously [28]. In the previous study the initial non-linear region occurred over a greater force and deformation range (Fig. 7), which may be due to the nominally larger mean scalp thickness (3.7 ± 1.8 mm vs 7 ± 1 mm [28]) and the non-uniformly distributed loading from the hemispherical anvil. The 3 m/s impacts in the current study exhibited a comparable stiffness (3850 ± 1201 N/mm) to those impacted at 7–8 m/s (3953 ± 986 N/mm [28]). However, this finding contradicts the rate-dependent nature of the human head’s response observed in the present study and reported by others [13, 28]. In the previous study, the heads tolerated a larger load (8716 ± 2083 N vs 12004 ± 2378 N [28]), and absorbed more energy (12.7 ± 3.4 J vs 25.3 ± 12.6 J [28]), prior to failure. Only cranial vault fractures were produced in the previous study, likely indicating that the concentrated stresses at the vertex (via the hemispherical anvil) were larger than the distributed stresses in the occipital region. Whereas in the present study, only skull base fractures were produced, probably because the occipital stresses were greater due to the smaller mount surface area that restricted contact to the occiput, compared to Yoganandan et al.’s [28] mount that contacted the entire skull base. The skull base fractures produced in the current study have been produced in cadaveric near-vertex head-neck impacts [15] and are observed clinically in falls thought to have a high probability of inversion [7], which suggests that the flat impactor and custom mount in this study provided a boundary condition with relevance to head-first impact cervical spine trauma.

Stiffness and peak force increased with velocity between 1 and 2 m/s (non-destructive), but there was no difference in these parameters between 2 and 3 m/s impacts. The rate dependency at lower velocities was consistent with Loyd et al. [13] who reported higher stiffness and peak force in non-destructive head impacts performed at 2.4 m/s, compared to 1.7 m/s. The rate independence observed at higher velocities was congruent with destructive studies [3, 5] performed at > 5 m/s in which peak force was not affected by velocity (stiffness was not evaluated).

The variation in mechanical outcomes across the specimens, for each impact velocity, was similar to those observed in previous vertex and parietal impact studies [1, 28]. These previous studies attributed the variation to physiological difference between humans. In this study, BMD and/or cranial height influenced the peak force, peak deformation and stiffness, but their effect was dependent on impact velocity. Greater cranial height was associated with higher peak force and lower deformation at 2 m/s, and greater stiffness at 3 m/s. Higher BMD was associated with increased stiffness at 2 m/s. There was no association detected at 1 m/s for either of these adjustment factors. As the cranial height represents the dimension of the head in the direction of the loading, and BMD is related to the elastic modulus of bone [20], it is possible that cranial height and BMD influenced the response of the head at each impact velocity, and this effect may be detected with greater specimen numbers.

With increasing loading rate, isolated cranial bone samples exhibit increased ultimate strength and elastic modulus, but reduced strain to failure and energy to failure [29]. In the non-destructive tests (1 and 2 m/s), peak deformation and absorbed energy were larger at 2 m/s than at 1 m/s impact velocity, commensurate with the fourfold increase in impactor energy at the higher velocity. In contrast, at 3 m/s impact, velocity fracture occurred at a lower deformation, likely because the bone had a lower energy absorption capacity at the increased loading rate. These data suggest that the relationship between loading rate, skull stiffness, energy to failure, and fracture response, is complex and should be considered when characterizing and modelling the impact response of the head.

The bilinear force-deformation response observed in the non-destructive tests has been previously described for the head [5, 28], but the mechanics underlying this observation are not clear. Sequential fracture of the outer then inner cortices of the skull has been hypothesized as a possible cause [5, 28], but fractures were not observed on post-impact CT of the specimens in the current study that exhibited bilinear response curves. A bilinear response has been observed during dynamic compression of isolated cortex-diploe-cortex skull samples [2], where the first linear region corresponded to elastic loading of the bone construct, the transition between linear regions coincided with trabecular collapse, and the second linear region corresponded to compression of trabecular debris between the inner and outer cortices. The transition between linear regions observed at 1 and 2 m/s could correspond to the production of microscopic fractures in the trabecular diploe, which were not observed on the CT scans due to insufficient image resolution.

Few ex vivo studies have observed clinically relevant skull base fractures resulting from head-first impacts [8, 15]. These injuries have only been produced in whole or head-neck cadavers [15], and not in isolated heads [27]. The skull base fracture load reported in the present study is likely non-biofidelic, due to the study design. As this study focused on characterizing the isolated head response rather than investigating the production of skull base fractures, a rigid occipital support was employed, which removed the physiological articulation of the atlanto-occipital joint. Furthermore, the mount area (2021 mm^2^) was larger than the articular area of the atlanto-occipital joint (359 mm^2^) [14], as it was not possible to further reduce the area and maintain secure support of the head during the impact event. The PETG mount likely led to a higher occipital fracture load tolerance, than if support was limited to only the atlanto-occipital joint, by distributing the load over a larger support area at the skull-mount interface. Lastly, the severity of the observed fractures may have been influenced by subsequent impacts as the carriage rebounded on the specimen until it came to a rest. Therefore, the presented model is likely not suitable for predicting skull base fracture tolerance in head-first impacts.

The present study characterized the force-deformation response of the head with boundary conditions approximating the second mode of near-vertex head-first impact, where the head is compressed at the vertex and occiput. For inverted cadaver head-neck drop tests (~3 m/s, 16 kg torso mass) [18], the peak head load during mode 2 was 3312 ± 991 N. Within this range of axial head force, the isolated cadaveric head was less compliant in the present study (flat impactor, ~ 3 m/s) compared to Yoganandan et al. [28] (hemispherical impactor, 7–8 m/s). In a near-vertex head-first impact model, a headform tuned to the response of the Yoganandan et al. [28] would be more compliant, which may influence the cadaveric neck’s kinetic response and the occurrence of cervical spine injuries, similar to the findings in head-first impacts onto padded versus rigid surfaces [19]. These findings reinforce our assertion that data from the current study is specific to the applied loading conditions (flat impact surface, 1–3 m/s). Additional biomechanical data may be required to validate headforms for vertex impacts with other loading conditions, such as oblique impacts, greater impactor energy (mass and/or velocity), and focal impact surfaces.

Due to limitations with specimen availability, two specimens (IDs 4 and 6) in the 1 m/s impact group had pre-existing facial fractures which were considered minor and unlikely to contribute substantially to specimen response. The pre- and post-test CT scans were compared and it was confirmed that the fractures had not propagated after testing. For both specimens the outcomes were within one standard deviation, except stiffness which was within 1.1 standard deviation, of the mean. Their individual force-deformation responses closely approximated the response corridor for the entire 1 m/s group (Supplementary Figure S4). The potential influence of these existing fractures was considered to be less than that of general inter-specimen variability.

A limitation of using cadaveric heads is the absence of cerebrospinal fluid and intracranial pressure, and the presence of air pockets in the cranial vault, which may alter the applied internal cranial vault boundary condition and change the structural response of the head, compared to the in vivo response. In this study, the cranial vault was effectively sealed by the PETG mount and PMMA, whereas in vivo fluid and neural tissue can displace through the foramen magnum. These differences in cranial vault content, pressure and sealing, likely alter the internal boundary condition of the ex vivo head compared to in vivo. This may affect skull deformation response, but to our knowledge no studies have specifically measured these effects. By leaving the intracranial contents intact, and not adding other fluid, gel or solid materials, our techniques are consistent with most studies that have measured human head stiffness [27].

The PETG mount, which was necessary to apply neck-end boundary conditions at the occiput, was not infinitely stiffer than the heads and may have contributed to the force-deformation response reported for the isolated cadaveric heads. If the measured stiffness is assumed to result from the combined stiffness of two springs (head and mount) in series, then it was estimated that the isolated stiffness of the head would increase by less than 11% of the mean stiffness for each impact velocity (Supplementary Methods).

The current study provides the structural response of the head when subjected to superior-inferior impacts by a 16 kg carriage with a flat impactor plate, at 1, 2 and 3 m/s. Impact velocity influenced peak force, deformation at peak force, stiffness and absorbed energy, as well as the presence of skull base fractures. These data can be used to develop surrogate head models with biofidelic responses to head-first impacts. Future work may investigate the underlying mechanisms of the observed bilinear force-deformation response and the production of skull base fractures with appropriate loading damping that replicates the loads transferred through the cervical spine during a head-first impact.

### Supplementary Information

Below is the link to the electronic supplementary material.

Supplementary file1 (PDF 398 kb)

## Data Availability

The data that support the findings of this study are publicly available in the Figshare repository, as part of this record: 10.25909/c.6823773 [31].
