# Supplementary material for: The Structural Response of the Human Head to a Vertex Impact
Source: Ann Biomed Eng. 2023 Sep 21;51(12):2897–907. doi: 10.1007/s10439-023-03358-z (PMC10632295; doi:10.1007/s10439-023-03358-z)
Supplement: Supplementary file 1 — Supplementary file1 (PDF 398 kb) [file 10439_2023_3358_MOESM1_ESM.pdf]

SUPPLEMENTARY MATERIAL

Supplementary Methods: Impact response of the custom head mounts

Custom head mounts were designed to support the head whilst approximating the boundary conditions at the skull base by the spinal column. The geometry of the mount was derived from a head-neck CT scan and generalized such that it would be adaptable for other skull bases (Figure S1). These mounts were 3D printed in polyethylene terephthalate glycol (PETG) filament with 100% infill. To verify that the mount's response was sufficiently stiff for cadaveric impact testing, isolated mount impact tests were performed.

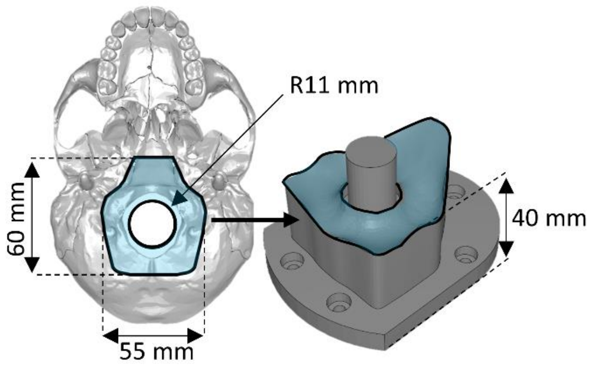

Figure S1: Inferior view of skull base with mount outline. The head is supported by the mount only in the blue shaded region.

Six mounts that were equivalent to those used in the study were 3D printed (polyethylene terephthalate glycol filament, 100% infill; Zortrax, Olsztyn, Poland). A removable support was temporarily adhered to the upper border of the mount, and then a layer of polymethylmethacrylate (PMMA; Kulzer, Hanau, Germany) was poured onto the mount to provide a flat impact surface. The mount was attached to a uniaxial load cell (66 kN; FFL-SPKT, Strainsert, Pennsylvania, USA) at the base of a custom drop tower (Figure S2A). Each mount was impacted by a 16 kg carriage at 1 or 2 m/s (N = 3 per impact velocity). The stiffness of the mounts was not characterized for 3 m/s due to safety concerns.

Loads and carriage position data were collected at 50 kHz using a data acquisition system (BNC-2120 & PXIe-4331, National Instruments, USA) and custom LabVIEW code (2019, National Instruments, Texas, USA). Mount deformation was defined as the displacement of the encoder (±10 µm resolution; LM15, Rotary and Linear Motion Sensors, Komenda, Slovenia) from the onset of contact. Data were processed using custom MATLAB code (R2020a, Mathworks, Massachusetts, USA). A fourth-order, two-way, low-pass Butterworth filter, with a 4 kHz cut off frequency, was applied to the data. The loading region of interest (ROI) was defined from the onset of contact (100 N increase in force) to 15 kN (Figure S2B); this ROI was selected as the loads were comparable to the loads experienced by the mount and head in the cadaveric testing. Stiffness was calculated as the slope of a linear regression fit from 3 to 15 kN.

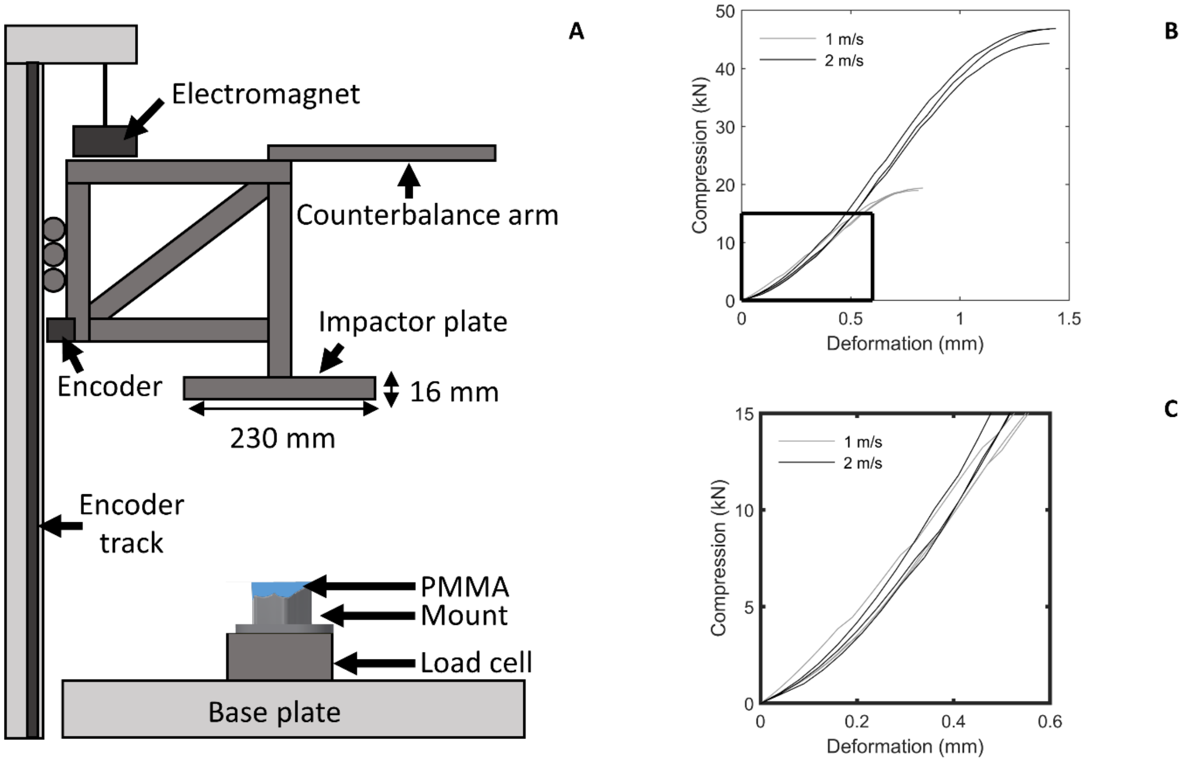

Figure S2: (A) Annotated illustration of drop tower apparatus impacts for impacting the mount and polymethylmethacrylate (PMMA). (B) Force-deformation response from the onset

of contact to maximum force for 1 and 2 m/s impact. (C) Force deformation response for 1 and 2 m/s impact trimmed to 15 kN.

The mount did not fail during the initial impact, or subsequent rebound events, for any of the six tests. Within the ROI, the force-deformation response for both impact velocities exhibited an initial toe-region, observed on high-speed footage as initial misalignment between the carriage and PMMA, followed by a quasilinear region. Across all tests the mount stiffness was 39,334 ± 8,382 N/mm. These findings indicate that the mount was approximately 10 times stiffer than the mean stiffness for 3 m/s cadaveric head impacts and approximately 13-16 times stiffer than the mean stiffness for 1 m/s and 2 m/s cadaveric head impacts.

Supplementary Tables:

Table S1: The force ($F_{transition}$) and deformation ($D_{transition}$) at the transition point between the two linear regions, and their respective stiffness values (k_1 and k_2), for specimens that exhibited a bilinear force-deformation response.

ID	$D_{transition}$ (mm)	$F_{transition}$ (N)	k_1 (N/mm)	k_2 (N/mm)
1	2.54	3555	1851	970
2	0.97	3239	4317	1550
3	1.26	3004	3652	1458
4	1.4	2967	2949	1675
5	1.17	2965	3626	1359
6	1.43	2384	2326	1457
7	1.34	2745	3032	1481
8	1.14	4102	4670	617
9	0.77	3555	5292	1178
10	0.9	3511	4678	1591
11	2.41	5422	2808	709
12	2.4	3810	1904	497
13	1.62	6891	5475	1401
14	1.93	4822	2877	829
16	1.76	7589	5221	629

Table S2: Final general linear models for peak force (N), peak deformation (mm), stiffness (N/mm) and absorbed energy (J).

**Peak Force**		
**Variable**	**Estimate (95% CI)**	**p-Value**
Velocity * cranial height		0.030
1 m/s	-290.3 (-529.2, -51.3)	0.020
2 m/s	-82.0 (-334.4, 170.4)	0.501
3 m/s [a]	-	-
[a] indicates reference category		
**Deformation**		
**Variable**	**Estimate (95% CI)**	**p-Value**
Velocity		0.003
1 m/s	-0.02 (-0.90, 0.86)	0.965
2 m/s	1.496 (0.56, 2.43)	0.002
3 m/s [a]	-	-
[a] indicates reference category		
**Stiffness**		
**Variable**	**Estimate (95% CI)**	**p-Value**
Velocity *BMD		<0.001
1 m/s	-0.605 (-12.3, 11.09)	0.919
2 m/s	25.24 (9.56, 40.91)	0.002
3 m/s [a]	-	-
Velocity *cranial height		<0.001
1 m/s	-210.55 (-330.53, -90.56)	0.001
2 m/s	-272.86 (-431.58, -144.14)	0.001
3 m/s [a]		
[a] indicates reference category		
**Energy**		
**Variable**	**Estimate (95% CI)**	**p-Value**
Velocity		<0.001
1 m/s	-4.93 (-9.19, -0.66)	0.024
2 m/s	9.32 (4.56, 14.10)	<0.001
3 m/s [a]	-	-
[a] indicates reference category		

Table S3: Post-hoc comparisons between impact velocities in the final general linear models for peak force (N), peak deformation (mm), stiffness (N/mm) and absorbed energy. Reported differences are between estimated marginal means for each level of impact velocity (B) and the reference (A).

	Velocity (A)	Velocity (B)	Mean Difference I-J (95% CI)	p-Value
**Force**	1	2	-2478.6 (-4218.3, -738.9)	0.005
		3	-3578.4 (-5278.9, -1877.7)	<0.001
	2	3	-1099.7 (-3014.1, 814.6)	0.433
**Deformation**	1	2	-1.52 (-2.77, -0.26)	0.011
		3	-0.02 (-1.1, 1.06)	1.000
	2	3	1.50 (0.25, 0.26)	0.005
**Stiffness**	1	2	-1394.9 (-2753.47, 36.33)	0.042
		3	-1785.05 (-2942.19, -627.9)	0.001
	2	3	-390.15 (-1595.25, 814.95)	1.000
**Energy**	1	2	-14.26 (-19.47, -9.05)	<0.001
		3	-4.93 (-10.14, 0.28)	0.071
	2	3	9.33 (3.5, 15.15)	<0.001

Table S4: Fracture descriptions

Specimen ID	Fracture description
1 – 14, 16	No observable skull fractures.
15	Extensive skull base fractures.
17	Extensive vault fractures and skull base fractures.
18	Extensive skull base fractures.
19	Extensive skull base fractures.
20	Extensive skull base fractures.
21	Extensive skull base fractures. Occiput to temporal fracture.
22	Extensive skull base fractures.

Supplementary Figures:

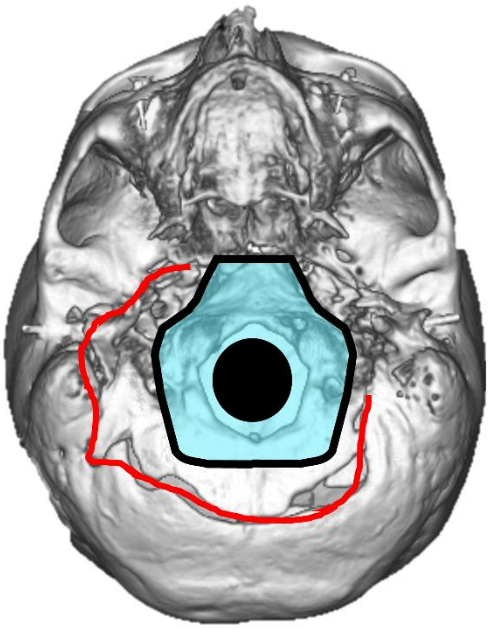

Figure S3: Exemplar fracture location (red line) from a 3D model of a post-test CT scan (ID: 17). The supporting area of the mount is highlighted (blue with black).

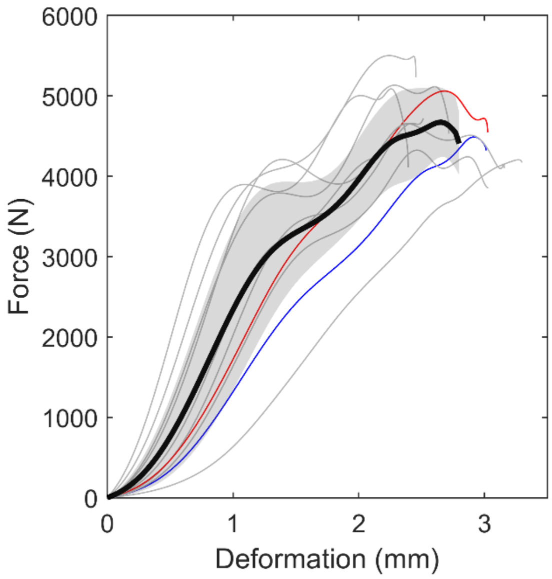

Figure S4: 1 m/s force-deformation responses. Specimens with existing fractures are colored (#4: red, #6: blue), remaining specimens are grey. Response corridor (±1 standard deviation; grey area) and characteristic average (black) are overlaid.
